# Exploring Lysine Incorporation as a Strategy to Mitigate Postsynthetic Halide Exchange in Lead-Halide Hybrid Perovskites

**DOI:** 10.1021/acsami.4c22194

**Published:** 2025-01-28

**Authors:** Arad Lang, Mariam Kurashvili, Johanna Sklar, Iryna Polishchuk, Awj Fada’os, Ithai Sessa, Altantulga Buyan-Arivjikh, Alexander Katsman, Jochen Feldmann, Boaz Pokroy

**Affiliations:** 1 Department of Materials Science and Engineering and the Russell Berrie Nanotechnology Institute, Technion−Israel Institute of Technology, Haifa 3200003, Israel; 2 Chair for Photonics and Optoelectronics, Nano-Institute Munich, Department of Physics, Ludwig-Maximilians-Universität (LMU), Königinstr. 10, Munich 80539, Germany; 3 Chair for Functional Materials, Department of Physics, TUM School of Natural Sciences, Technical University of Munich (TUM), James-Franck-Str. 1, Garching 85748, Germany; 4 The Nancy and Stephen Grand Technion Energy Program, Technion − Israel Institute of Technology, Haifa 3200003, Israel

**Keywords:** hybrid perovskite, halide exchange, migration, diffusion, amino acids, X-ray diffraction, bioinspiration

## Abstract

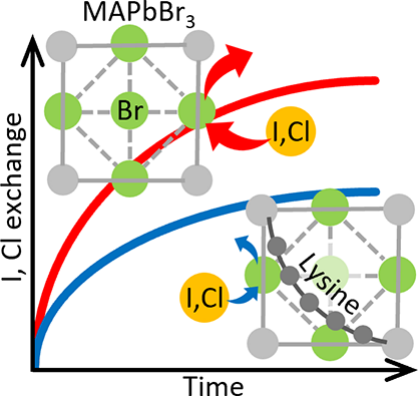

Lead-halide hybrid perovskites (RNH_3_PbX_3_, X = halide, e.g., Cl, Br, I; R = organic moiety) show promise for next-generation optoelectronic devices due to their simple synthesis routes, strong light absorption, and high photoluminescence quantum yield. However, postsynthetic halide exchange in lead-halide perovskites poses a challenge for the functionality of many perovskite devices. For example, in all-perovskite heterostructures, halide diffusion results in the formation of undesired mixed alloys rather than sharp interfaces required for many optoelectronic applications. To address this issue, we incorporated lysine molecules, one of the 20 common amino acids, into a hybrid perovskite MAPbBr_3_ (MA = CH_3_NH_3_) host and investigated their impact on the host’s ability to undergo postsynthetic halide exchange. We immersed lysine-incorporated MAPbBr_3_ crystals in solutions containing Cl^–^ or I^–^ for varying durations and analyzed subsequent halide exchange-related changes using ion chromatography, high-resolution powder X-ray diffraction, and photoluminescence spectroscopy. Our findings unanimously indicate that incorporated lysine significantly impedes postsynthetic Cl^−^ and I^−^ diffusion into bulk MAPbBr_3_. Our new bioinspired approach opens a route toward mitigating postsynthetic halide exchange in lead-halide hybrid perovskites and improving the suitability of perovskite devices for optoelectronic applications.

## Introduction

**Figure 1:**
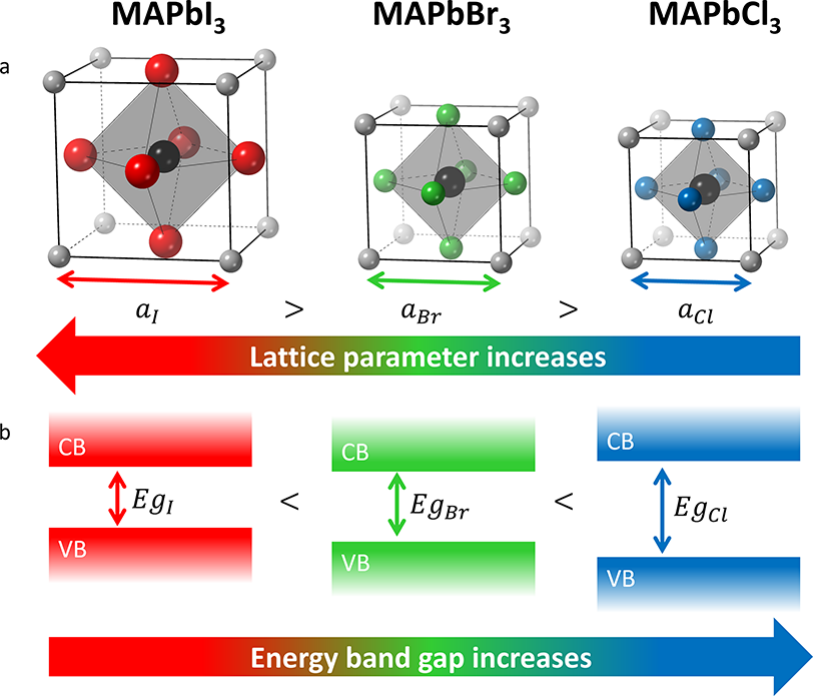
**MAPbX3 perovskites.** Schematic representations of (a) the unit cell, and (b) the band gap of MAPbX_3_ crystals. Ions are color-coded as follows: MA^+^ - gray; Pb^2+^ - black; I^–^ - red; Br^–^ - green; and Cl^–^ - blue. VB and CB denote the valence band and conduction band, respectively. For simplicity, the cubic structure of MAPbI_3_ (stable at high temperatures) is depicted.

Hybrid organic–inorganic perovskites (HOIPs) have been at the center of interest for the last few decades. This family of materials, characterized by the ABX_3_ perovskite structure, consists of dozens of different chemical compositions,^1−4^ with potential applications in optoelectronics,^5−7^ ferroelectricity,^8−10^ and magnetism.^11−13^ One of the most studied HOIPs, and the simplest from a structural point of view, is methylammonium lead halide – MAPbX_3_. It adopts a cubic (*Pm*3̅*m*, for MAPbCl_3_ and MAPbBr_3_)^14−16^ or a tetragonal (*I*4/*mcm* for MAPbI_3_)^14,15,17^ perovskite unit cell at room temperature, with methylammonium cations (CH_3_NH_3_^+^ = MA^+^) in its A-site (cell’s corners), Pb^2+^ cations in its B-site (cell’s center), and a halide (Cl^–^, Br^–^ or I^–^) in its X-site (faces’ centers) (Figure 1a). MAPbX_3_ holds tremendous potential for integration into optoelectronic devices, particularly solar cells,^18−23^ light-emitting diodes (LEDs),^24−28^ and high-energy radiation detectors.^29−31^

Lead halide perovskite crystals possess the unique ability to undergo rapid halide exchange.^32^ When immersed in a solution containing Y^–^ halides (where Y^–^ refers to a halide different than X^–^ as in the original crystal), the result is a solid solution in the form of APbY_*z*_X_3-z_. This process of halide exchange is diffusion-governed.^33^ Adjusting the halide ratio Y^–^/X^–^ (i.e., adjusting the stoichiometric coefficient *z*) allows for precise tuning of the crystals’ band gap across the entire visible light spectrum.^34−36^ In addition to altering optical properties, halide exchange induces changes in the crystal structure due to the different ionic radii of the halides,^37^ leading to changes in the lattice parameter (see Figure 1b). However, halide migration within crystals can negatively impact the performance of perovskite devices,^38−40^ and compromise the material’s crystallinity.^33^

Various strategies have been explored to mitigate halide exchange in perovskites. For example, the deposition of a thin layer of a nonperovskite material at the interface of all-perovskite heterostructure has been shown to reduce halide exchange.^38,41^ Similarly, defect passivation of the MAPbI_3_ layer using different amino acids in the perovskite solar cells has been reported to suppress I^–^ migration in the layer and improve device operation.^42,43^ Another approach involves partial substitution of the initial A-site cation with another material. For example, partially substituting MA^+^ cations into mixed formamidinium perovskites (FAPbBr_1.8_I_1.2_) suppresses photoinduced halide phase segregation.^44^ Alternatively, substituting MA^+^ with low concentrations of guanidinium molecule in MAPbI_3_ increases the activation energy for iodide diffusion, strongly reducing iodide transport through the perovskite.^45^ Similarly, partial substitution of MA^+^ by acetamidinium molecules induces lattice distortions that create a steric effect, impeding ion migration pathways and increasing iodide diffusion activation energy.^46^ These findings highlight that A-site cation engineering is a promising avenue for mitigating postsynthetic halide exchange in hybrid perovskites. Mastering and regulating halide exchange is essential for engineering efficient perovskite devices optimized for specific optoelectronic applications.

In this work, we present a novel approach of mitigating postsynthetic Cl^−^ and I^−^ exchange in bulk MAPbBr_3_ crystals by incorporating individual lysine (Lys) molecules *into* the MAPbBr_3_ crystalline lattice. The inspiration for incorporating amino acids into bulk semiconductors derives from nature, specifically from biominerals, which are organic–inorganic nanocomposites.^47−49^ For example, mollusk shells, composed of brittle calcium carbonate, are remarkably hard and tough due to (among else) intra- and intercrystalline organic inclusions.^49^ The incorporated molecules strongly influence the microstructure and crystal lattice of biogenic calcium carbonate single crystals.^50−52^ Drawing from this, we previously showed that inorganic semiconductor host crystals such as zinc oxide (ZnO),^53,54^ copper oxide (Cu_2_O),^55^ and lead sulfide (PbS),^56^ as well as the HOIP MAPbBr_3_,^57^ can incorporate individual amino acids. Through a combination of experimental techniques and theoretical calculations, we showed that amino acid incorporation can tune the optical and structural properties of these host semiconductors.^55,58,59^

In our earlier work,^57^ we successfully incorporated Lys into the MAPbBr_3_ crystal structure. The incorporation occurs when the two amine groups in Lys replace two MA^+^ cations along the ⟨110⟩ direction, forming a “molecular bridge”. The incorporation of Lys in MAPbBr_3_ leads to a contraction of the unit cell of the host crystal, alters the thermal expansion coefficient of MAPbBr_3_, and improves its stability under humid conditions.^57^ Since the MAPbBr_3_ crystals studied here were grown following the same procedure as in our previous work, we assume the Lys incorporation described earlier applies to this study as well. Furthermore, studies show that amino acids can affect the crystallization kinetics and preferred orientation of MAPbI_3_ crystals.^60,61^

To investigate the effect of Lys incorporation on the host’s propensity to undergo postsynthetic halide exchange, we synthesized Lys-incorporated MAPbBr_3_ crystals and immersed them in solutions containing Cl^–^ or I^–^ for specific durations to induce halide exchange. The resulting samples were then characterized chemically, structurally, and optically using ion chromatography (IC), synchrotron high-resolution powder X-ray diffraction (HR-PXRD), and photoluminescence (PL) spectroscopy.

## Experimental Section

### Crystal Synthesis

MAPbBr_3_ crystals were synthesized using a slow-grown method previously described in our work.^14,57^ In brief, 20 mL Pb^2+^ solutions in concentrated HBr, with varying amounts of Lys (*w* = 0, 1, or 2 gr), were heated to 95 °C. MAOH solution (also in HBr) was added, followed by natural cooling to room temperature. This process precipitated mm-sized orange MAPbBr_3_ crystals, which were then filtered, washed with acetone, and air-dried. The amount of incorporated Lys is proportional to the amount of Lys in the synthesis.^57^

### Diffusion Experiments

Diffusion of halides into MAPbBr_3_ was performed following the procedure outlined by Osherov et al.^33^ Stock solutions of 0.1 M MAX (X = Cl, I) were prepared by dissolving MAX in isopropanol (iPrOH). Approximately 0.5 gr of Lys was added to each solution, causing it to become slightly cloudy (due to Lys being above its solubility limit in iPrOH). Approximately 500 mg of each MAPbBr_3_ sample was added to a 15 mL centrifuge tube. Then, 10 mL of MAX solution was added using a syringe and a 0.45 μm cellulose filter to ensure saturation with Lys without any undissolved aggregates. The tubes were sealed and placed on a rocking stage in the dark for specific time periods (*t* = 1, 6, and 24 h). Subsequently, the samples were filtered using Whatman No. 5 filter paper, washed three times with acetone, and dried in air.

### Halide Determination

10 mg of each sample were transferred to a polypropylene tube, followed by the addition of 10 mL of DI water. The tubes were sealed and placed on a rocking stage for 48 h, until complete dissolution. Then, the samples were diluted by taking 1 mL of each and adding 9 mL of DI water. Since the concentrationof I^-^ in the solution is small, its determination was performed without the final dilution step. The determination of anions was employed using ion chromatography (IC), using the instrument 881 Compact IC pro – Anion-MCS, with chemical suppression by the Metrohm Suppressor Module (MSM), equipped with 836 Compact Autosampler (Metrohm AG, Switzerland). Anion separation was done on Shodex column IC-SI-52 4E (4.0 mmID x 250 mmL). 3.6 mM sodium carbonate (Na_2_CO_3_) solution was applied as an eluent.

### Structural Characterization

HR-PXRD was performed in ID22 of the European Synchrotron Radiation Facility (ESRF), Grenoble. France. Several mg of each sample were ground and placed inside a borosilicate capillary, which was sealed using hot wax and placed on a brass holder. To avoid beam damage, each sample underwent three fast scans, which were later averaged. Data processing was performed as follows: First, we fitted the (100) reflection of each sample to a Voigt function,^62^ using the OriginLab software, and extracted the diffraction peak position (2θ_(100)_). Then, we calculated the lattice parameters of our (cubic) samples according to Equation 1:
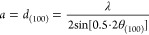
1where *d*_*(100)*_ is the interplanar spacing of the (100) planes, and λ is the X-ray wavelength (λ = 0.3542 Å), and calculated the lattice distortion according to Equation 2:
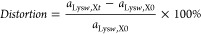
2where *a*_Lys*w,*X*t*_ is the lattice parameter of the sample grown with *w* concentration of Lys in solution (*w* = 0, 1, 2 gr) and after diffusion of halide X for *t* hours (*t* = 0, 1, 6, 24).

To assess the inhomogeneity of the samples based on the asymmetry of the (100) diffraction peaks, we calculated the centroid of each peak according to Equation 3:
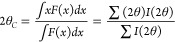
3where *I(2*θ) is the diffraction intensity measured at angle *2*θ. Then, we defined the inhomogeneity measure as the relative difference between the peak Bragg position (*2*θ_*B*_) and its centroid (*2*θ_C_), i.e.:
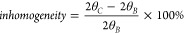
4

The results of 2θ_*B*_ and 2θ_*C*_ for all (100) reflections are summarized in Table S1.

### PL Spectroscopy

Steady-state PL measurements were performed on a homemade μ-PL setup. Depending on the excitation wavelength, pulsed white light generated by SuperL Extreme EXR-20 Supercontinuum White Light Laser (NKT Photonics) was directed into either to SuperK Extend-UV unit (NKT Photonics) (380 nm) or to an acousto-optic tunable unit SuperK Select (NKT Photonics) (485 nm). The laser excitation pulse width was τ_*pulse*_≈ 20 ps. The excitation laser beam was guided to the sample with help of mirrors. A dichroic mirror (DM) (Semrock) reflected the laser beam at a 90*°* angle toward an objective (Olympus SLCPLFL 40*x*/0.55) that focused it onto the sample. The excitation laser spot diameter was approximately 1 μm. The DM was selected in such a way, that only the PL of the sample was transmitted but not the laser beam reflected back from the sample. The Acton SpectroPro SP2300 spectrometer and a charge coupled device camera PIXIS 400 eXcelon, both from Princeton Instruments were used to record the PL spectra. An additional long pass filter (Thorlabs) in front of the spectrometer was used to filter out the residual laser light that was able to go through the DM. MAPbBr_3-x_Cl_*x*_ samples were excited at 380 nm (3.26 eV), *f*_*rep*_*=* 26 MHz with an average excitation power <0.3 μW, whereas MAPbBr_3-y_I_*y*_ samples were excited at 485 nm (2.56 eV), *f*_*rep*_*=* 1.22 MHz, with an average excitation power <3.9 μW. The excitation power was kept as low as possible and the PL spectra were detected immediately after excitation to avoid any effects due to light-induced halide segregation.^63,64^

### SEM-EDS

Energy dispersive X-ray spectroscopy was performed using a Zeiss Ultra+ scanning electron microscope. The samples were glued to a stainless-steel stub holder using carbon tape, and coated with a few nm of carbon to improve their electrical conductivity. The energy of the primary electron beam in the SEM was set to 10 keV.

## Results and Discussion

We synthesized MAPbBr_3_ crystals with different amounts of incorporated Lys using the procedure described by Lang et al.^14,57^ Subsequently, we immersed the crystals in a solution containing methylammonium halide (MACl or MAI) for different periods of time, following the method outlined by Osherov et al.^33^ (for details see Experimental Section). The resulting MAPbCl_*x*_Br_3-x_ and MAPbI_*y*_Br_3-y_ crystals are referred to as **Cl***t* and **I***t*, respectively, where *t* = 1, 6, 24 is the immersion duration, in hours. The reference MAPbBr_3_ crystals with *t* = 0 are denoted as ref. *x* and *y* are the stoichiometric coefficients of the exchanged Cl^−^ and I^−^, respectively, with 0 ≤ *x,y* ≤ 3. Additionally, the samples with different Lys concentrations are referred to as **Lys***w*, where *w* = 0, 1, 2 is the added mass of Lys in the initial crystallization solution, in grams.

**Figure 2:**
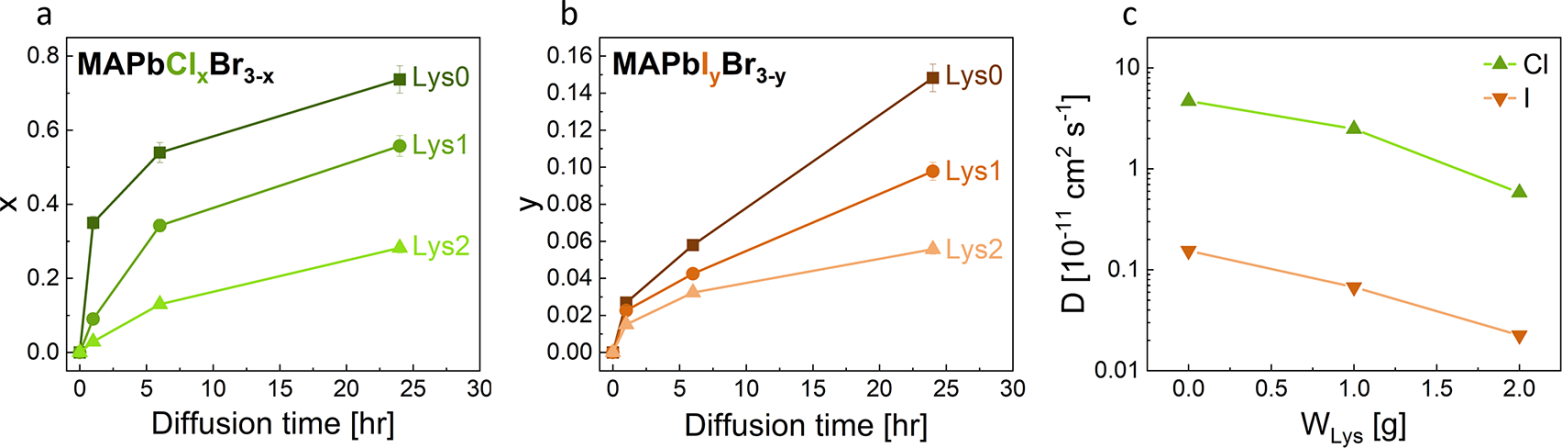
**Diffusion analysis via ion chromatography.** The stoichiometric coefficients *x* and *y* of (a) MAPbCl_*x*_Br_3-x_ and (b) MAPbI_*y*_Br_3-y_ at different concentrations of incorporated Lys as a function of immersion time in MACl and MAI solutions, respectively. (c) Calculated diffusion coefficients of Cl^−^ and I^−^ in Lys-incorporated MAPbBr_3_.

To determine the stoichiometric coefficients of halides in mixed halide MAPbCl_*x*_Br_3-x_ and MAPbI_*y*_Br_3-y_ crystals, we employed IC. Figure 2a,b presents the measured values of *x* and *y* as functions of the immersion time of MAPbBr_3_ crystals with different amounts of incorporated Lys in a solution containing methylammonium halide. As expected, longer immersion times result in higher values of *x* and *y* for Cl^−^ and I^−^ diffusion, respectively. For the same immersion duration, *x* is consistently higher than *y,* reflecting the higher diffusion coefficient of Cl^−^ compared to I^−^ due to variances in their ionic radii.^33,37^ Notably, the data reveals that for a fixed immersion time, samples with incorporated Lys have lower *x* and *y* values than those without incorporated Lys. Specifically, after 24 h of immersion, *x* and *y* values of the samples with the highest Lys concentration (Lys2) are 62% lower compared to the sample with no Lys (Lys0), as shown in Figure 2. This finding suggests that the incorporated Lys is hindering halide exchange. Such inhibitory effect persists even after prolonged immersion times (see Figure S1).

To evaluate the reduction of halide exchange in Lys-incorporated MAPbBr_3_, we modeled the halide exchange process as the diffusion of halides into a spherical MAPbBr_3_ crystal, assuming the halide concentration at the crystals’ surface to be constant.^65^ Using this model, the diffusion coefficients of Cl^−^ and I^−^ within MAPbBr_3_ (denoted *D*_Cl_ and *D*_I_, respectively) can be calculated using Equation 5:
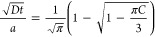
5

This approach provides a quantitative measure to assess how Lys incorporation affects the diffusion of halides within MAPbBr_3_ crystals. Here, *a* represents the radius of the sphere (set as *a* = 250 μm, see Figure S5), *t* is the diffusion time, and *C* denotes the average Cl^−^ or I^−^ concentration within the sphere, which is proportional to *x* and *y*, respectively (for details see Supplementary Note 2). The calculated diffusion coefficients, as a function of the mass of added Lys, are shown in Figure 2c. Lys incorporation leads to a substantial reduction in both *D*_Cl_ and *D*_I_ values. As expected, due to the difference in ionic radii, the diffusion coefficient of Cl^−^ in MAPbBr_3_ is significantly higher than that of I^−^ (*D*_Cl_ ≫ *D*_I_).^33^ The impact of Lys on both coefficients is comparable, with their calculated values decreasing by approximately 10-fold upon incorporation (see Figure S8).

Halide exchange within HOIPs is a diffusion-controlled process that occurs through a substitution mechanism. For halide exchange to take place, the diffusing halide must have nearby vacancies and sufficient thermal energy to migrate.^66^ Hence, the diffusion coefficient depends on both vacancy formation (*v*) and halide migration (*m*) and can be expressed as
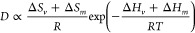
6where Δ*S* and Δ*H* respectively represent the changes in entropy and enthalpy associated with vacancy formation and halide migration, *T* is the absolute temperature, and *R* is the ideal gas constant. When considering the effects of Lys incorporation, it is reasonable to assume that it primarily influences the enthalpy changes, thereby impeding halide diffusion by increasing Δ*H*_*v*_ and Δ*H*_*m*_. This effect is attributed to the C–Br interactions formed during the slow growth of Lys-incorporated MAPbBr_3_,^57^ which hinder the formation of Br vacancies and act as physical barriers to halide migration within the crystal. As a result, the activation energy required for halide diffusion is increased, thereby reducing the efficiency of halide substitution. This phenomenon aligns with previous findings in the literature, where partial substitution of A-site cations was shown to impede halide transport.^44−46^

**Figure 3:**
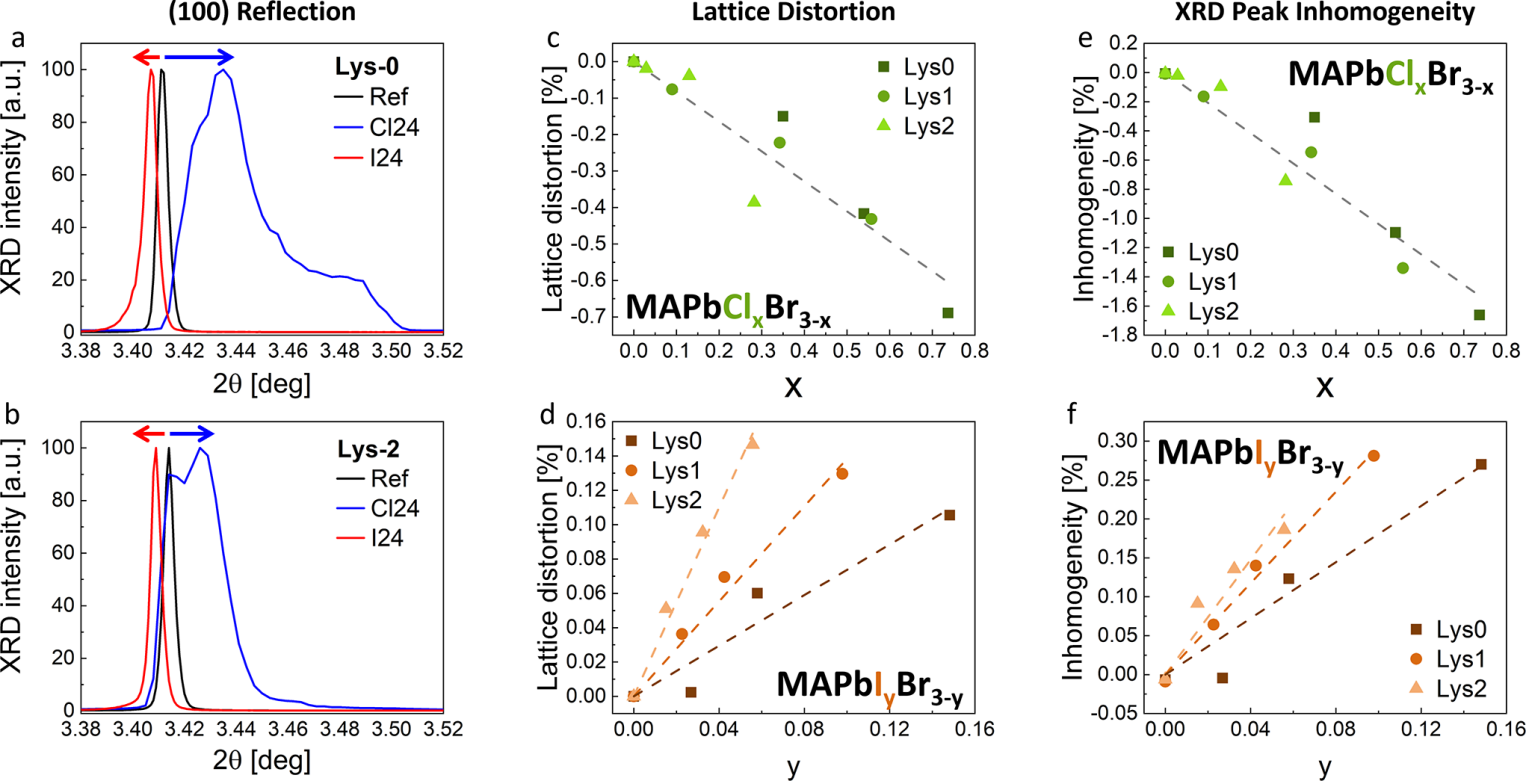
**Effect of incorporated Lys on halide exchange-related structural changes. (a + b)** The (100) HR-PXRD reflection of reference MAPbBr_3_ (black), MAPbCl_*x*_Br_3-x_ (blue), and MAPbI_*y*_Br_3-y_ (red) after 24 h diffusion, without (*top*) and with (*bottom*) incorporated Lys. **(c + d)** Lattice distortion, calculated based on HR-PXRD peak positions, of MAPbCl_*x*_Br_3-x_ (*top*) and MAPbI_*y*_Br_3-y_ (*bottom*). **(e + f)** Inhomogeneity, calculated based on HR-PXRD peak asymmetry, of MAPbCl_*x*_Br_3-x_ (*top*) and MAPbI_*y*_Br_3-y_ (*bottom*). Dashed trend lines serve as a guide for the eye. Diffraction patterns were collected at a wavelength of 0.3542 Å.

To evaluate whether the Lys-induced reduction in halide exchange correlates with diminished halide exchange-associated structural changes, we conducted HR-PXRD analysis. All samples maintained a cubic perovskite structure (*Pm*3̅*m*), with some samples exhibiting noticeable diffraction peak broadening (see Figure S2). While MAPbI_3_ typically adopts a trigonal unit cell at room temperature (*I4/mcm*),^14,17^ this structure was not observed in any of the I-exchanged samples, likely due to the low I^−^ concentration (low *y* values). Consistent with our previous results,^57^ Lys incorporation in MAPbBr_3_ crystals induces lattice contraction (see Figure S3). The zoom-in on the (100) XRD reflection is shown in Figure 3a,b. For Cl^−^ exchange, diffraction peaks shift toward higher *2*θ values, while for I^−^ exchange, they shift toward lower angles, indicating lattice contraction and expansion, respectively (also compare Figure 1).

To assess the impact of Lys on halide exchange-related structural changes, we calculated the lattice distortion (see Experimental Section). The results are illustrated in Figure 3c,d. For Cl^−^ exchange (Figure 3c), lattice distortion is negative, indicating lattice contraction.^14,37^ As expected for a solid solution, the Cl-induced lattice distortion exhibits linear relationship with *x,* with overlapping distortion curves across different incorporated Lys concentrations (the gray dashed line in Figure 3c). Interestingly, the magnitude of Cl^−^ exchange-related lattice distortion is significantly reduced for the sample with the highest level of Lys incorporation, confirming that the incorporated Lys mitigates postsynthetic Cl^−^ diffusion in MAPbBr_3_ crystals.

For I^−^ exchange (Figure 3d), the lattice distortion is positive, indicating lattice expansion, consistent with the larger size of I^−^ compared to Br^−^.^14,37^ The lattice distortion in the I-exchanged samples is linear in *y*. However, unlike Cl-exchanged samples, the I-exchanged samples show distinct linear trends with varying slopes for different Lys concentrations. This behavior likely arises from the opposing effects of I^−^ and Lys on the crystal structure: while Lys incorporation into MAPbBr_3_ crystals induces lattice contraction,^57^ exchanging Br^−^ with I^−^ causes lattice expansion.^14,33^ The lattice distortion appears to depend not only on the amount of exchanged I^−^ but also on the degree to which the lattice of the MAPbBr_3_ crystal was already contracted by the incorporated Lys (for details see Supplementary Note 1). Figure 3c,d shows that the magnitude of lattice distortion induced by I^−^ exchange is less pronounced than that induced by Cl^−^ exchange, in accordance with IC measurements (*y* < *x*, see Figure 2). The reduced amount of exchanged I^−^ compared to Cl^−^ might also contribute to this effect being relatively prominent in MAPbI_*y*_Br_3-y_ samples.

A closer examination of the HR-PXRD patterns in Figure 3a,b (and Figure S2) reveals that halide exchange not only shifts the diffraction peaks but also causes their noticeable broadening and increased asymmetry. This asymmetric broadening is most pronounced in the Lys-free sample (Lys0) after the longest Cl^−^ diffusion time (Cl24) (Figure 3a). The broadening, indicative of crystal inhomogeneity, arises from the diffusion-controlled halide exchange process, creating a halide concentration gradient from the crystal surface toward its core, which directly impacts the crystal structure. Since HR-PXRD measures an ensemble of thousands of unit cells, the resulting signal represents a superposition of individual XRD signals from regions with varying Cl^−^ concentrations (see Figure S4).

To assess the effect of incorporated Lys on the crystal inhomogeneity, we calculated the center of mass of the diffraction peaks (centroid) and defined an inhomogeneity measure as the difference between the diffraction peak position and its centroid (for details see Experimental Section). The results are presented in Figure 3e,f. In the case of MAPbCl_*x*_Br_3-x_, inhomogeneity becomes more negative (increases in magnitude, see Figure 3e) as more Cl^−^ is exchanged. Conversely, for MAPbI_*y*_Br_3-y_, inhomogeneity increases in magnitude as well, but it becomes more positive (Figure 3f). For Cl^−^ exchange, inhomogeneity exhibits a linear relationship with *x*, similar to the lattice distortion trend shown in Figure 3c. Importantly, samples with higher concentrations of incorporated Lys show reduced inhomogeneity, indicating hindered Cl^−^ diffusion. On the other side, for I^−^ exchange, the inhomogeneity follows a linear trend with *y*, but displays distinct linear behaviors with varying slopes for different Lys concentrations, mirroring the lattice distortion trends shown in Figure 3d. For the same value of *y*, samples with more Lys show slightly greater asymmetry of the diffraction peaks. Overall, confirming the inhibition of I^−^ exchange due to Lys incorporation using the HR-PXRD measurements is less straightforward compared to Cl^−^ exchange and necessitates an alternative method for further investigation.

A complementary approach to investigate the effect of the incorporated Lys on the halide exchange is through optical spectroscopy. Optical studies have demonstrated that substituting one halide for another modifies the electronic band structure of the perovskite, resulting in a change in its band gap.^35,67^ Changes in the band gap of a solid solution as a function of its composition are often described by a second-order polynomial function.^67−70^ However, at low substitution concentrations, such as those in this work, the modification in the band gap is well approximated by a linear function.^67,71^ Substituting Br^−^ with Cl^−^ in MAPbBr_3_ crystals induces a blue-shift (increase) in the band gap, whereas substituting Br^−^ with I^−^ causes a red-shift (decrease) (Figure 1). The extent of the band gap shift is directly correlated with the magnitude of halide exchange.^35,72^ The change in PL spectra of the mixed halide perovskites has been shown to closely follow the trend of their band gap shifts.^67,71^ Due to the highly scattering nature of the samples investigated in this work, conventional transmission UV–vis spectroscopy cannot be used to measure absorbance and assess their band gaps. However, the extent of halide exchange can be evaluated by comparing the PL spectra of halide-exchanged samples to the reference Lys-incorporated MAPbBr_3_ spectrum.

**Figure 4:**
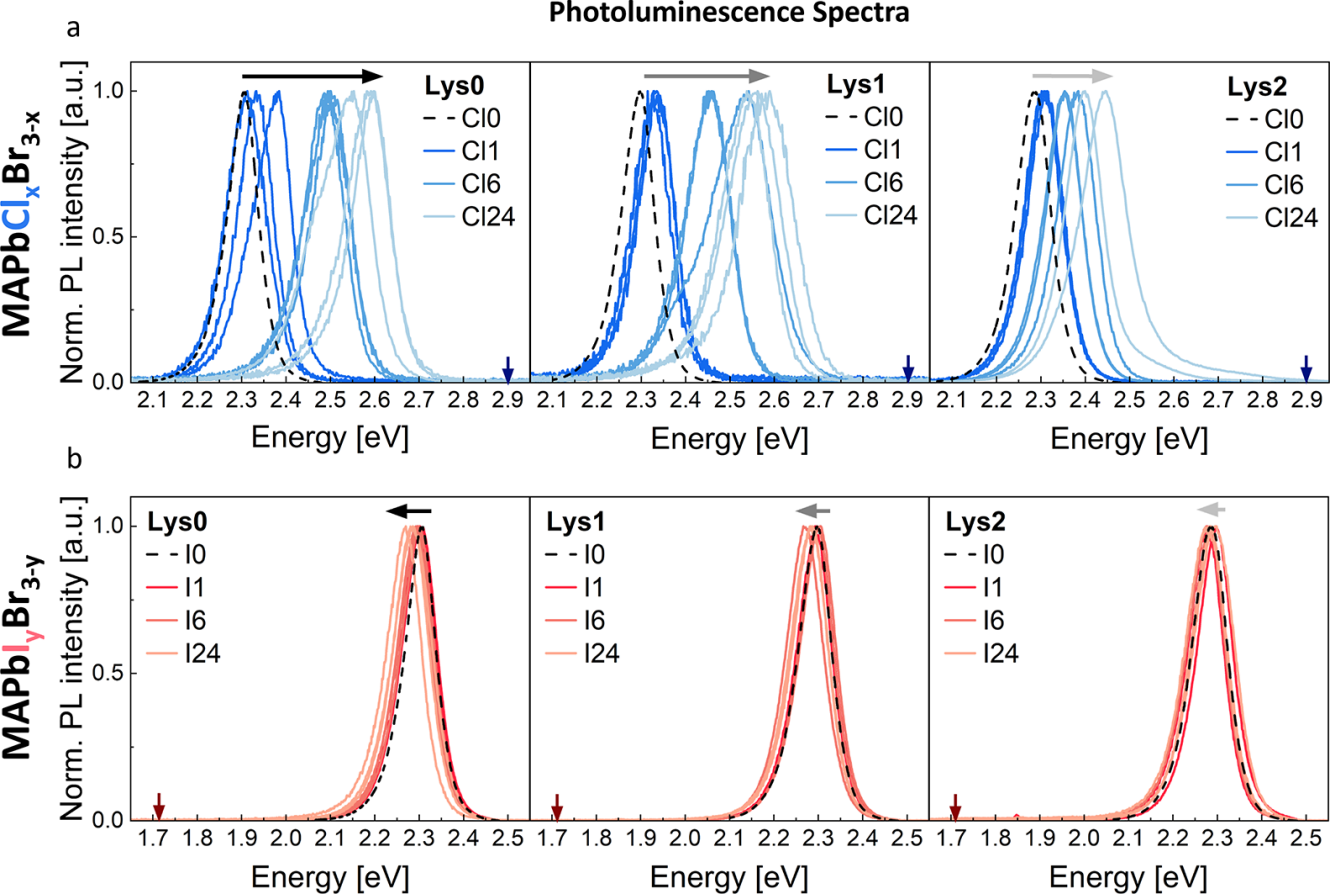
**Photoluminescence spectra for different amounts of incorporated Lys**. PL spectra of (a) MAPbBr_3-x_Cl_*x*_, and (b) MAPbBr_3-y_I_*y*_ samples. In each panel, multiple peaks of the same color represent measurements taken at different spots on the same sample. The black dashed curves represent the PL spectra of the respective reference MAPbBr_3_ crystals, without Cl^−^ or I^−^. The incorporated Lys content increases from left to right. The dark blue and red arrows denote the approximate energetic position of the PL peaks of bulk MAPbCl_3_ and MAPbI_3_, respectively.

Figure 4a,b displays PL spectra of MAPbCl_*x*_Br_3-x_ and MAPbI_*y*_Br_3-y_ at different incorporated Lys concentrations, measured at multiple spots, and recorded immediately after the optical excitation. For a better comparison, the spectra are normalized at maximum intensity. For Cl^−^ exchange (Figure 4a), longer immersion times of MAPbBr_3_ in the MACl solution result in a stronger blue-shift of the PL spectra toward the energetic position of the PL peak of pure MAPbCl_3_ (marked with blue arrows in Figure 4a).^73,74^ Importantly, the magnitude of this shift decreases with higher incorporated Lys content, indicating hindrance of Cl^−^ exchange.

Both the energetic position and the shape of the PL spectra of the mixed-halide MAPbCl_*x*_Br_3-x_ are spot-dependent. With a light penetration depth of a few tens to hundreds of nm in MAPbBr_3_ crystals for the chosen excitation energy,^73^ and focused beam diameters around 1 μm, PL spectroscopy probes only a fraction of the micron-sized crystals. Each spectrum is representative of a small section of the whole crystal (see Figure S5**)**. Broadening of the PL spectra following Cl^−^ exchange can be attributed to the compositional inhomogeneity of the measured spot (see Figures S6a, S7a). Different regions of the crystal have varying Cl^−^ concentrations, leading to local variations in halide content. These variations translate into different local band gaps, as the band gap is a function of halide composition. Excitons that diffuse into regions with lower band gaps during their lifetime emit lower-energy photons, while those confined to higher band gap regions are unable to migrate and instead emit higher-energy photons. This process leads to a more pronounced inhomogeneous broadening of the PL spectra in halide-exchanged samples compared to the uniform MAPbBr_3_ crystals. Hence, this result points toward an inhomogeneous Cl^−^ distribution within the perovskite crystals, consistent with the asymmetric (100) diffraction peak in Figure 3a and inhomogeneity in Figure 3e.

Similarly, Figure 4b depicts the PL spectra of MAPbI_*y*_Br_3-y_ crystals. With prolonged immersion time of MAPbBr_3_ crystals in the MAI solution, the PL spectra of the resulting samples red-shift toward the energetic position of the PL peak of pure MAPbI_3_ (marked with red arrows in Figure 4b).^68,73^ Most importantly, we can clearly see that the magnitude of this shift decreases with higher incorporated Lys concentration, becoming almost undiscernible for the samples with the highest Lys content (Lys2). This finding indicates that incorporated Lys mitigates I^−^ exchange. The magnitude of the I-related red-shift is considerably smaller compared to the Cl-related blue-shift, consistent with the IC data in Figure 2. Similar to Cl^−^, the PL spectrum of MAPbI_*y*_Br_3-y_ is spot-dependent and exhibits broadening (see Figures S6b, S7b), indicating an uneven distribution of I^−^ within the crystals.

**Figure 5:**
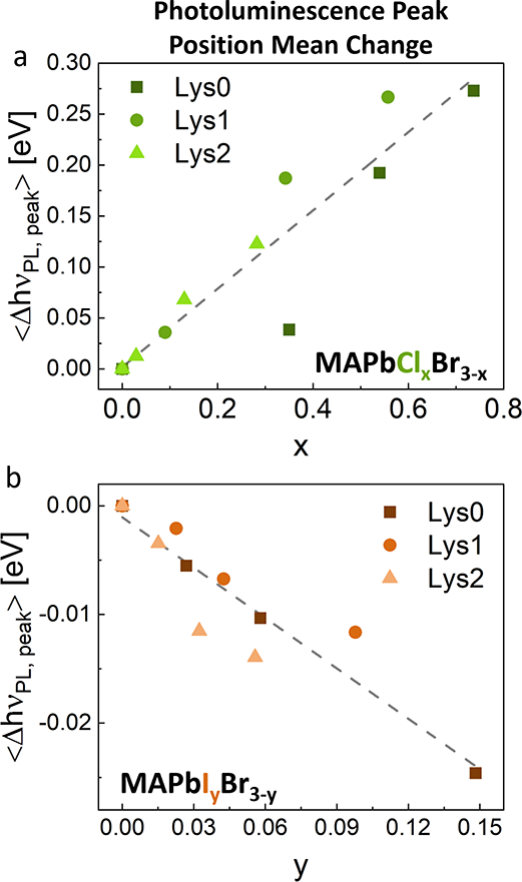
**Change in the photoluminescence peak position.** Mean change in the energetic position of the PL peak for (a) MAPbCl_*x*_Br_3-x_ and (b) MAPbI_*y*_Br_3-y_, calculated relative to the PL peak position of the reference MAPbBr_3_. The gray dashed lines serve as a guide for the eye. The errors in ⟨Δ*h*ν_PL,peak_⟩, taken as the standard deviation between different measured values, are all smaller than the data points.

To quantify the changes in PL due to halide exchange, we have calculated the mean shift in the PL peak energy of the halide-exchanged crystals relative to the average PL peak energy of the respective Lys-incorporated MAPbBr_3_. These data for MAPbCl_*x*_B_3-x_ and MAPbI_*y*_Br_3-y_ are plotted in Figure 5a,b, respectively. In both cases, the relative mean PL peak position increases linearly (in magnitude) with growing *x* and *y*, as expected.^72^ The observed linear trend is a direct indication that Lys incorporation hinders postsynthetic halide exchange of both Cl^−^ and I^−^, since the magnitude of the energetic shift of the PL is directly proportional to the exchanged halide content. This finding aligns with the observations made using IC and XRD.

## Conclusions

In summary, our findings unanimously indicate that incorporated lysine impedes postsynthetic Cl^−^ and I^−^ diffusion into bulk MAPbBr_3_ and underscore the significant impact of molecular inclusions and A-site cation engineering on the physical properties of crystalline hosts. The incorporation of Lys into the host MAPbBr_3_ affects the latter’s ability to undergo postsynthetic halide exchange, as evidenced by IC. Higher levels of incorporated Lys lead to a reduction in the exchanged halide content for a given immersion time of MAPbBr_3_ crystals in a solution containing methylammonium halide (Cl^−^ or I^−^). The hindrance of the postsynthetic Cl^−^ and I^−^ diffusion into Lys-incorportaed MAPbBr_3_ crystals is further supported by theoretical modeling of the diffusion coefficients of the halides.Similarly, HR-PXRD analysis confirms this for MAPbCl_*x*_Br_3-x_ samples, where higher levels of incorporated Lys show less halide diffusion-related structural changes, indicating reduced Cl^−^ exchange. For MAPbI_*y*_Br_3-y_, the structural changes are not only dependent on the exchanged I^−^ content but also determined by the incorporated Lys, making detangling the effect of Lys on the structural changes more difficult. PL spectroscopy further confirms these results, demonstrating that the samples with higher Lys content exhibit a reduced PL shift related to Cl^−^ and I^−^ exchange, indicating an overall slower exchange process.

While this study focuses on MAPbBr_3_, the underlying mechanism by which Lys inclusions hinder halide diffusion is likely applicable to other perovskite systems. This approach could similarly mitigate postsynthetic halide exchange in both other hybrid perovskites (e.g., FAPbI_3_) and fully inorganic perovskites (e.g., CsPbBr_3_). Additionally, molecular inclusions within bulk perovskite crystals hold potential for addressing photoinduced halide segregation, a phenomenon that reduces open-circuit voltage in mixed-halide perovskite solar cells.^75−77^ To achieve substantial levels of molecular incorporation—resulting in significant structural and physical changes to the host material—careful selection of the incorporated molecules and precise adjustment of synthetic procedures are essential, as we have previously demonstrated. Our new bioinspired approach could be leveraged to mitigate undesired halide diffusion in perovskite-based devices.

## Supporting Information Available

Supporting Information including a discussion regarding the proposed diffusion models, full analysis of the HR-PXRD and PL peaks, SEM-EDS elemental maps, and long-time diffusion IC results (PDF)
