# Supplementary material for: Exploring Lysine Incorporation as a Strategy to Mitigate Postsynthetic Halide Exchange in Lead-Halide Hybrid Perovskites
Source: ACS Appl Mater Interfaces. 2025 Jan 28;17(6):9485–93. doi: 10.1021/acsami.4c22194 (PMC11826507; doi:10.1021/acsami.4c22194)
Supplement: Supplementary file 1 — am4c22194_si_001.pdf [file am4c22194_si_001.pdf]

Supporting Information

Exploring Lysine Incorporation as a Strategy to Mitigate Post-Synthetic Halide Exchange in Lead-Halide Hybrid Perovskites

Arad Lang[1], Mariam Kurashvili[2], Johanna Sklar[1], Iryna Polishchuk[1], Awj Fada'os[1], Ithai Sessa[1], Altantulga Buyan-Arivjikh[3], Alexander Katsman[1], Jochen Feldmann[,2] and Boaz Pokroy[**,1,4]*

[1] Department of Materials Science and Engineering and the Russell Berrie Nanotechnology Institute, Technion−Israel Institute of Technology, 3200003 Haifa, Israel.

[2] Chair for Photonics and Optoelectronics, Nano-Institute Munich, Department of Physics, Ludwig-Maximilians-Universität (LMU), Königinstr. 10, 80539 Munich, Germany.

[3] Chair for Functional Materials, Department of Physics, TUM School of Natural Sciences, Technical University of Munich (TUM), James-Franck-Str. 1, 85748 Garching, Germany

[4] The Nancy and Stephen Grand Technion Energy Program, Technion − Israel Institute of Technology, Haifa 3200003, Israel.

*feldmann@lmu.de.

**bpokroy@technion.ac.il.

Supplementary Notes

Supplementary Note 1: Diffraction peak shift

Let us define a_{Lys} as the lattice parameter of the Lys-incorporated $MAPbBr_3$. Assuming small linear changes, we can write:

$$(S1) \quad a_{Lys} = a_0 + m_{Lys}w$$

where a_0 is the lattice parameter of pure $MAPbBr_3$, w is the mass of Lys added to the solution, and $m_{Lys} < 0$ (lattice contraction). Similarly, for the halides exchange, we can write:

$$(S2) \quad a_{Lys\_Cl} = a_{Lys} + m_{Cl}x \,; \qquad a_{Lys\_I} = a_{Lys} + m_I y$$

When a_{Lys_Cl} and a_{Lys_I} are the lattice parameters of the samples after Cl or I exchange, respectively, x, y are the stoichiometric coefficients, $m_{Cl} < 0$ (contraction), and $m_I > 0$ (expansion).

Combining equation (S1) and (S2) gives:

$$(S3a) \quad a_{Lys\_Cl} = a_0 + m_{Lys}w + m_{Cl}x$$

$$(S3b) \quad a_{Lys\_I} = a_0 + m_{Lys}w + m_I y$$

Note, that by doing so we assume that the two causes for lattice distortion (Lys incorporation *or* halide exchange) are decoupled, i.e., they do not affect one another. We used the measured (experimental) lattice parameters of the samples that did not undergo halide exchange (i.e., Lys0-Cl0, Lys1-Cl0, and Lys2-Cl0) to calculate m_{Lys}. Moreover, we used the lattice parameters of the samples containing no Lys to calculate m_{Cl} and m_I (Lys0-Cl0, Lys0-Cl1, Lys0-Cl6, and Lys0-Cl-24 for m_{Cl}, and Lys0-Cl0, Lys0-I1, Lys0-I6, and Lys0-I24 for m_I). The fitting is presented in **Figure S9** and its results are summarized in **Table S2**.

Next, we used these values to calculate the expected lattice distortions on the rest of the samples, containing both Lys *and* Cl or I (**Figure S10**). It appears, that for $MAPbCl_xBr_{3-x}$ (**Figure S10**a) our analysis is accurate,

as the measured lattice distortion follows the calculated trend, regardless of the concentration of incorporated Lys. However, the situation is different for $MAPbI_yBr_{3-y}$ (**Figure S10**b) – our calculations only describe the Lys0 case.

This anomaly makes us to revise our previous assumption regarding the decoupling of the Lys and I effects. Hence, we now assume that for a certain concentration of exchanged I, its effect on the lattice parameter depends also on the concentration of incorporated Lys. In other words, for the case of I exchange, the causes for lattice distortion are coupled. Assuming this coupling has a linear relation, we can write:

$$(S4) \quad m_I = m_{I0} + nw$$

Where m_{I0} is the value of m_I with no incorporated Lys, and n is the slope. We can find m_{I0} and n by plotting the slopes of the lattice parameters vs. y of different Lys concentrations, followed by fitting it to a linear trendline (**Figure S11**).

Finaly, using both Equation (*S3b*) and Equation (*S4*) we get:

$$(S5) \quad a_{Lys\_I} = a_0 + m_{Lys}w + (m_{I0} + nw)y$$

Equation (*S5*) indeed suggests a different linear trend for each Lys concentration. **Figure S12** demonstrates how it fits to the experimental data.

Supplementary Note 2: Diffusion modelling

According to Carslaw and Jaeger (ref. 74 in the main text), the average concentration of the diffusing species within a sphere of radius *a* at time *t* is given by:

$$(S6) \quad \frac{C}{C_s} = \frac{6\sqrt{Dt}}{a\sqrt{\pi}} - \frac{3Dt}{a^2} + \frac{12\sqrt{Dt}}{a}\sum_{n=1}^{\infty}\left[\frac{1}{\sqrt{\pi}}e^{-\frac{n^2a^2}{Dt}} - \frac{na}{\sqrt{Dt}}erfc\left(\frac{na}{\sqrt{Dt}}\right)\right],$$

where C_s represents the constant concentration of the diffusing species at the surface of the sphere, *D* is the diffusion coefficient, and *erfc*(*x*) denotes the complementary error function. *C* denotes the average Cl or I concentration within the sphere, which is proportional to *x* and *y*, respectively.

In our case ($D_{max} \approx 10^{-10}$, $t_{max} = 24\ hr, a = 250\ \mu m$), we find that $\sqrt{Dt}/a \approx 0.1 < 0.5$. Therefore, the third term in the solution can be neglected, simplifying the equation to:

$$(S7) \qquad \frac{\sqrt{Dt}}{a} = \frac{1}{\sqrt{\pi}}\left(1 - \sqrt{1 - \frac{\pi C}{3C_s}}\right)$$

Let us define the following terms:

$$(S8) \qquad f(C) \equiv 1 - \sqrt{1 - \frac{\pi C}{3C_s}} \qquad k \equiv \frac{\sqrt{\pi D}}{a}$$

Assuming $C_s = 1$, we can rewrite Eq. (S7) as follows:

$$(S9) \qquad f^2(C) = k^2 t$$

Based on the IC data (**Figure 2** in the main text), we can plot $f^2(C_{Cl})$ and $f^2(C_I)$ against diffusion time for various Lys concentrations, when *C* is the atomic concentration of the exchanged halide:

$$(S10) \qquad C_{Cl} = \frac{x}{3} \qquad C_I = \frac{y}{3}$$

These plots are then fitted to a linear trend, as described by Eq. (S9), with the slope representing k^2. The fitted results are shown in **Figure S13**, and the corresponding data is summarized in **Table S3**. The high R^2 values confirm the model's suitability for our experimental results. The only exception is the diffusion of Cl^- in Lys0 samples. Given that this scenario involves the most advanced diffusion (i.e., the highest concentration of exchanged halide), it is possible that additional factors, such as surface effects, must be considered.

Supplementary Tables

Table S1. Fitting results for the HR-PXRD experiment

		$MAPbCl_xBr_{3-x}$		$MAPbI_yBr_{3-y}$	
W_{Lys}	Time	$2\theta_B$	$2\theta_C$	$2\theta_B$	$2\theta_C$
[g]	[hr]	[deg]	[deg]	[deg]	[deg]
**0**	0	3.4115	3.4118	3.4115	3.4118
**0**	1	3.4167	3.4271	3.4115	3.4116
**0**	6	3.4258	3.4634	3.4095	3.4053
**0**	24	3.4352	3.4923	3.4079	3.3987
**1**	0	3.4127	3.4130	3.4127	3.4130
**1**	1	3.4153	3.4209	3.4114	3.4092
**1**	6	3.4203	3.4390	3.4103	3.4055
**1**	24	3.4275	3.4734	3.4083	3.3987
**2**	0	3.4140	3.4142	3.4140	3.4142
**2**	1	3.4146	3.4153	3.4122	3.4091
**2**	6	3.4153	3.4186	3.4107	3.4061
**2**	24	3.4272	3.4527	3.4090	3.4026

Table S2. Calculated values for *m* parameters.

Parameter	Calculated value
$\boldsymbol{m_{Lys}}$	-0.0021
$\boldsymbol{m_{Cl}}$	-0.0488
$\boldsymbol{m_{I}}$	+0.0438

Table S3. Calculated k^2 values and coefficient of determination.

	Cl diffusion		I diffusion	
w_{Lys}	k^2 (*10^{-4})	R^2	k^2 (*10^{-4})	R^2
**0**	8.491	0.906	0.279	0.988
**1**	4.487	0.986	0.121	0.995
**2**	1.051	0.996	0.041	0.991

Supplementary Images

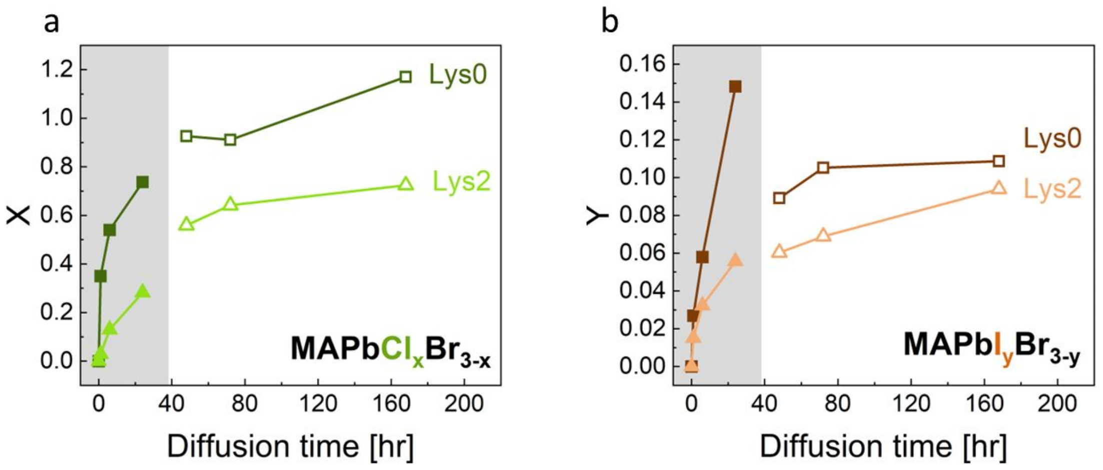

Figure S1. IC results for (a) Cl and (b) I diffusion. Filled data points (grey background) represent the short-time diffusion experiment (as shown in Figure 2), while empty data points (white background) correspond to long-time diffusion experiment on freshly synthesized $MAPbBr_3$-Lys samples. Due to possible differences in incorporated Lys concentration and the crystallite size between the initial and newly prepared samples, quantitative comparison between short- (initial) and long-term (new) experiments is difficult but qualitative trends remain valid. For Cl, the amount of alloyed Cl is significantly lower in the Lys-incorporated sample (Lys2) comparted to the Lys-free sample (Lys0) even after a week of diffusion. For I, the amount of alloyed I is consistently lower in the Lys2 sample comparted to Lys0 sample, but after one week of diffusion, the I concentrations in both samples become nearly identical, likely due to loss of crystallinity induced by prolonged I diffusion. Such a change reduces the crystallite size, effectively accelerating the diffusion process.

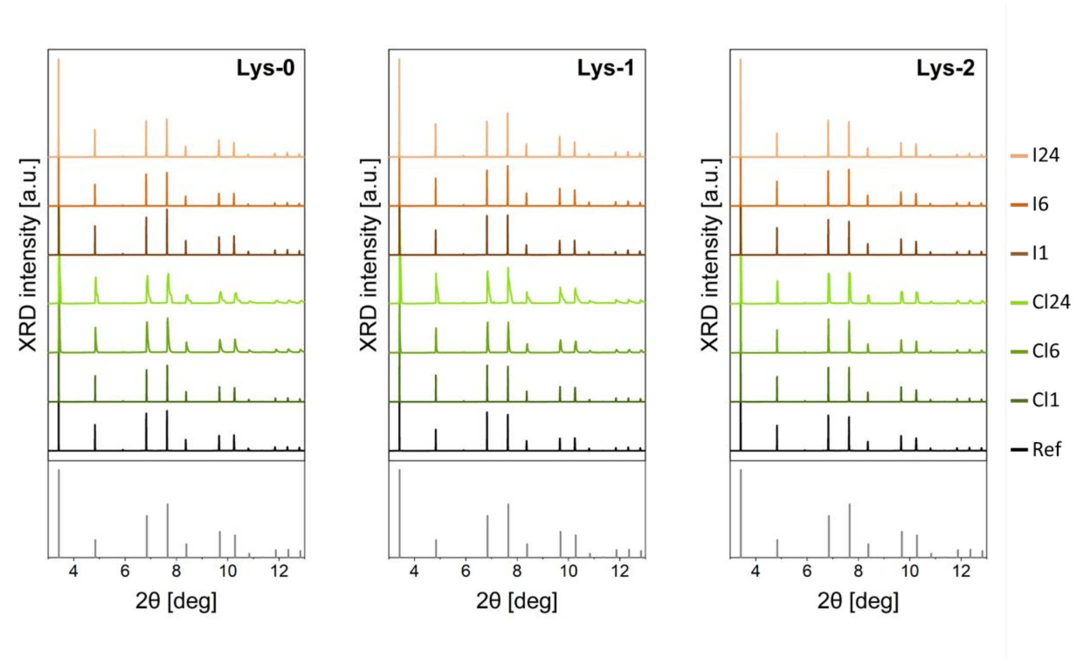

Figure S2. Comparison between $MAPbBr_3$ reflections (according to CCDC Entry: SIYMUL) in grey lines, and the collected HR-PXRD data.

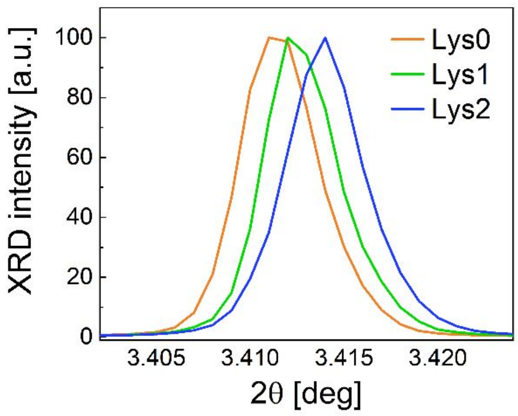

Figure S3. (100) reflection of the reference $MAPbBr_3$ samples (no exchanged halides), showing a gradual shift of the diffraction peak to higher Bragg angles with increasing Lys incorporation, indicating lattice contraction.

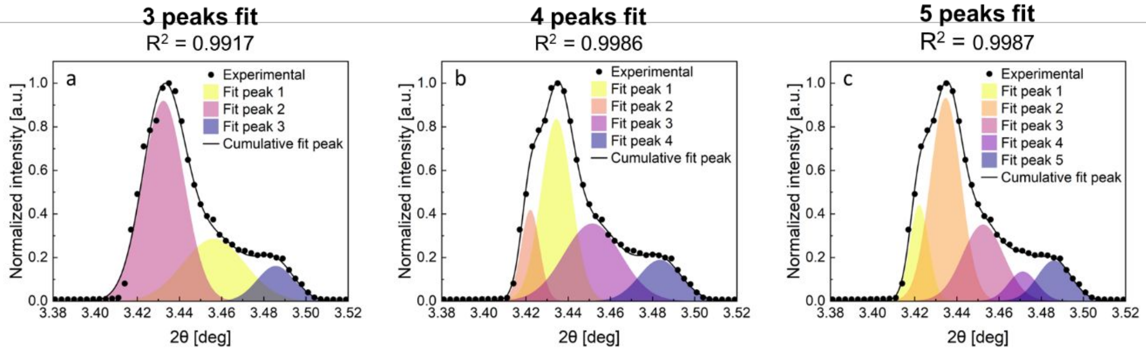

Figure S4. The asymmetric broadening observed in the HR-PXRD peaks, indicative of crystal inhomogeneity, arises from the diffusion-controlled halide exchange process, which directly impacts the crystal structure. Due to the continuous variation in halide concentration within the crystals, it is impractical to fully deconvolute the HR-PXRD peaks, as this would require an infinite number of components. Exemplary deconvolutions of the (001) HR-PXRD peaks for the Lys0-Cl24 sample are shown, using (a) 3, (b) 4, and (c) 5 peaks. Each fitted peak corresponds to $MAPbCl_xBr_{3-x}$ with a distinct x value.

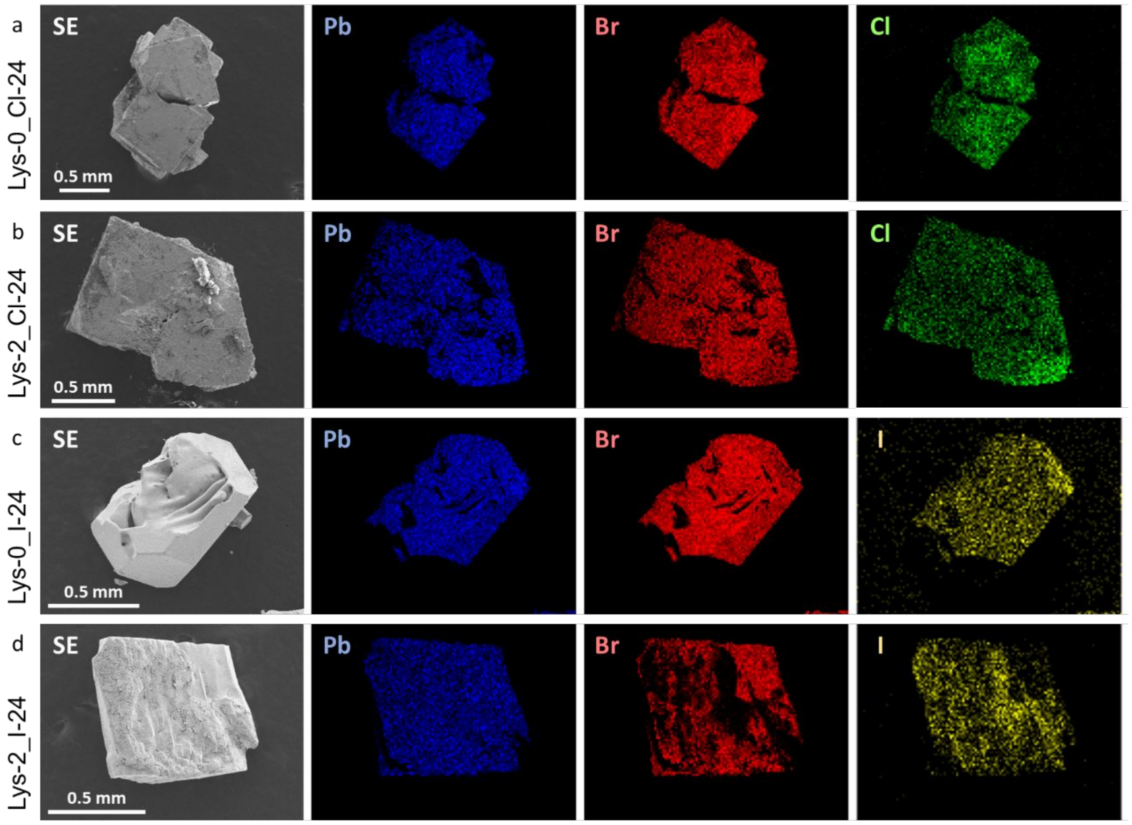

Figure S5. SEM micrographs and EDS elemental maps of selected samples, all after immersion in halide-containing solution for 24 hours: (a) $MAPbCl_xBr_{3-x}$ without incorporated Lys, (b) $MAPbCl_xBr_{3-x}$ with incorporated Lys, (c) $MAPbI_yBr_{3-y}$ without incorporated Lys, and (d) $MAPbI_yBr_{3-y}$ with incorporated Lys. The colors for the EDS signal are as follows: Pb - blue; Br - red; Cl - green, I - yellow. SE stands for secondary electrons, which were used to acquire the images.

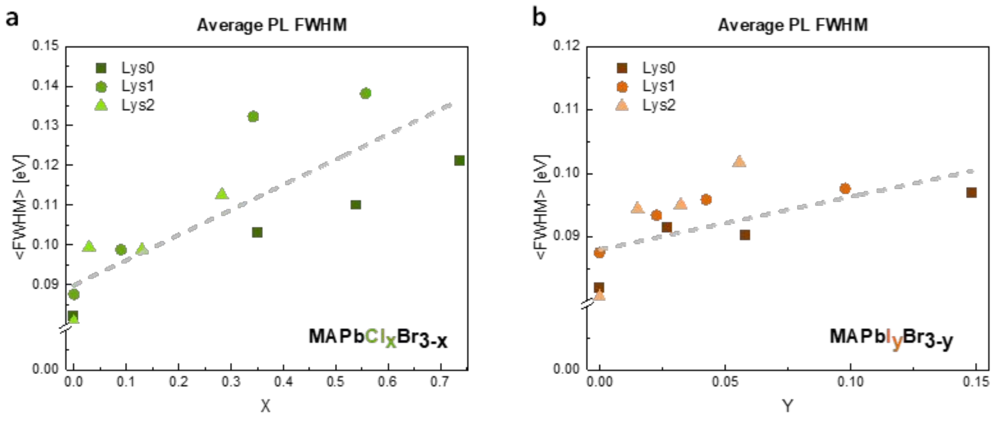

Figure S6. The average change in PL FWHM upon halide exchange for (a) $MAPbCl_xBr_{3-x}$ and (b) $MAPbI_yBr_{3-y}$ samples at varying levels of Lys incorporation. The PL spectra of halide-exchanged samples exhibit more pronounced inhomogeneous broadening compared to $MAPbBr_3$. For Cl-exchanged samples, the PL FWHM increases linearly with the stoichiometric number x. In contrast, for I-exchanged samples, the PL FWHM shows a slight increase with higher I content, but this trend does not appear to follow a linear relationship with the stoichiometric number y. Grey dashed lines are provided as visual guides.

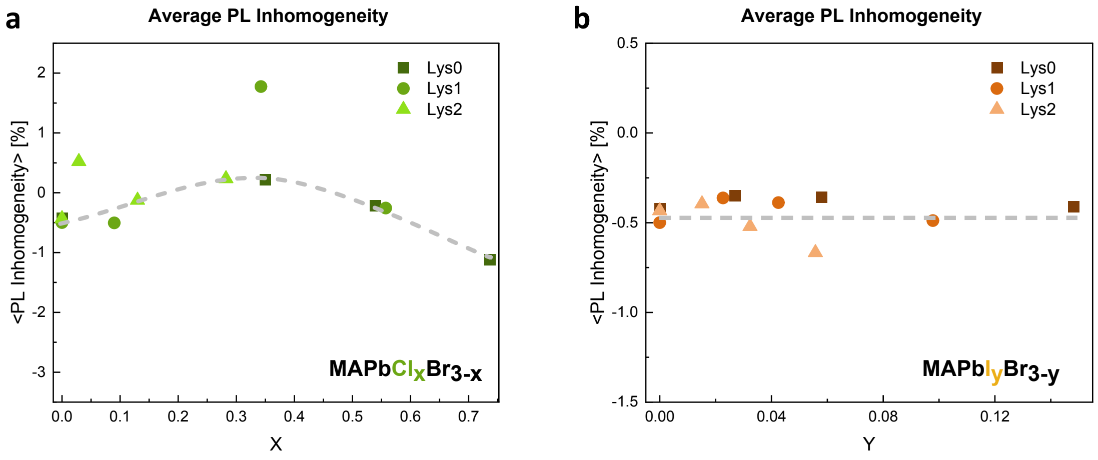

Figure S7. To evaluate the halide exchange-related inhomogeneous broadening of the PL spectra, we calculated the centroid of each peak, defined as: $\nu_C = \frac{\int xF(x)dx}{\int F(x)dx} = \frac{\sum \nu I(h\nu)}{\sum I(h\nu)}$, where, $I(h\nu)$ is the PL intensity at a given energy $h\nu$. We quantified the inhomogeneity as the relative difference between the peak PL emission energy $(h\nu_{PL})$ and its centroid $(h\nu_c)$: $PL\ inhomogeneity = \frac{h\nu_C - h\nu_{PL}}{h\nu_{PL}} \times 100\%$. PL inhomogeneities were calculated for (a) $MAPbCl_xBr_{3-x}$ and (b) $MAPbI_yBr_{3-y}$ crystals with varying lysine incorporation levels.

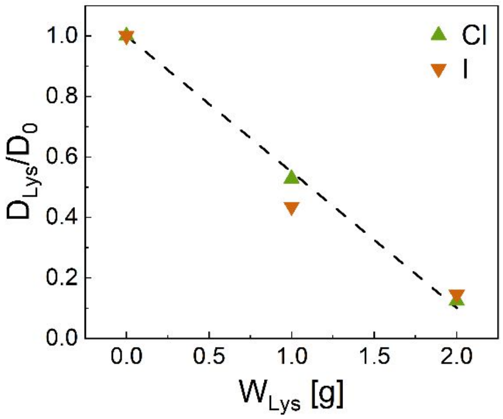

Figure S8. Change in diffusion coefficients for both Cl and I exchange due to Lys incorporation, calculated relative to the diffusion coefficients in the absence of Lys. The dashed black line indicate the shared linear trend (R^2=0.993).

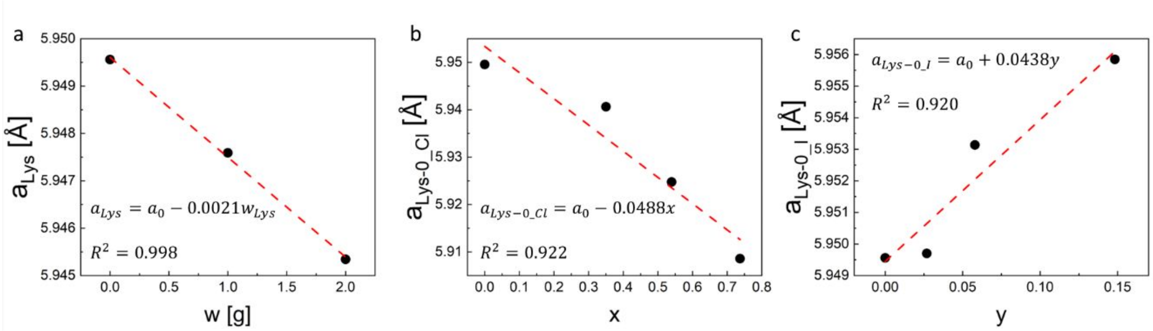

Figure S9. Calculations of (a) m_{Lys}, (b) m_{Cl}, and (c) m_I parameters.

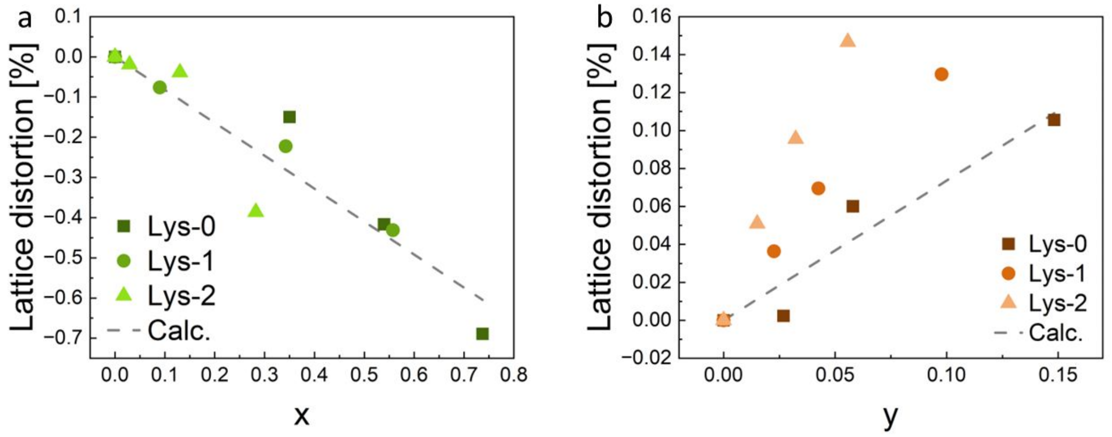

Figure S10. Expected lattice distortions (dashed grey lines) for (a) Cl, and (b) I exchange, calculated based on the values from Table S2.

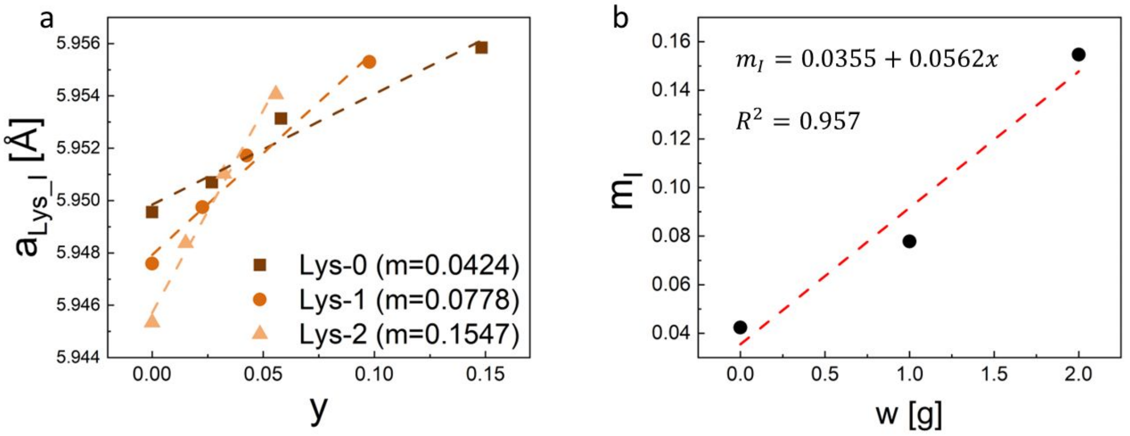

Figure S11. (a) Calculation of m_I values. (b) Extracted m_I values according to Equation (S4).

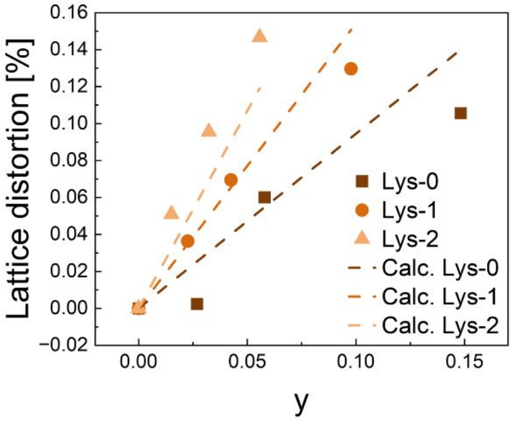

Figure S12. Expected lattice distortions (dashed lines), calculated based on the values from Figure S11.

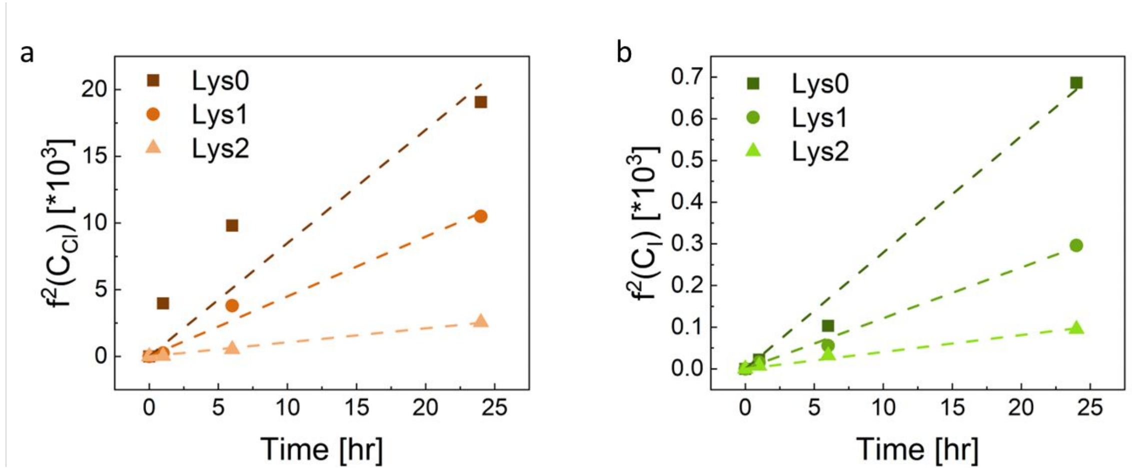

Figure S13. Calculations of k^2 (the slope of the dashed lines) for the diffusion of (a) Cl and (b) I in $MAPbBr_3$.
